# Plasma mitochondrial DNA is elevated in maternal serum at first trimester and may serve as a biomarker for prediction of gestational diabetes mellitus

**DOI:** 10.1111/1753-0407.13462

**Published:** 2023-09-01

**Authors:** Dushyant Kumar Sahu, Jessy Abraham

**Affiliations:** ^1^ Department of Biochemistry AIIMS‐Raipur Raipur India

## Abstract

**Background:**

We evaluated whether an abundance of first‐trimester plasma mitochondrial DNA (mtDNA) fragments could predict the risk for the development of gestational diabetes mellitus (GDM) by the late second or early third trimester.

**Methods:**

It was a prospective study wherein we enrolled 150 women in their first trimester of gestation. Oral glucose tolerance test (OGTT) was administered both in the first and second trimesters to diagnose GDM.

**Results:**

Among our cohort, 23 women were diagnosed with GDM in the first trimester and excluded from the study. Of the remaining 127, 29 women were diagnosed with GDM in the second trimester, and 98 women who did not develop GDM served as controls. We amplified blood drawn from each participant during the first trimester for three distinct mtDNA gene sequences: COX, ND4, and D‐loop. An abundance of each mtDNA sequence, estimated by the ΔCt method between mtDNA and 18S rRNA, correlated with GDM occurrence in the late second or early third trimester. There was a significant difference in ΔCt COX between controls and those with GDM occurrence in the second trimester (*p* = .006). These levels were not associated with age or fasting plasma glucose levels in the first trimester. ΔCt COX could predict GDM with a sensitivity of 90% and a specificity of 40%. Though ΔCt ND4 was higher in the GDM‐positive group, the levels did not reach statistical significance. ΔCt D‐loop was similar in GDM‐positive cases and controls who did not develop GDM during pregnancy.

**Conclusions:**

These results were in plasma samples collected 3 to 4 months before overt hyperglycemia diagnosis suggestive of GDM. The abundance of plasma mtDNA fragments represents a promising cost‐effective, convenient early‐stage biomarker for predicting GDM development. Importantly, it can be administered irrespective of the fasting status of the subject. Further assessment of the predictive capacity of these biomarkers within large, diverse populations is needed for effective clinical utility.

## Graphical abstract

**Highlights**

- In the first trimester, the plasma mitochondrial DNA (mtDNA) was higher in women who developed gestational diabetes mellitus (GDM) than in women who did not develop GDM during their pregnancy.
- The increased levels of plasma mtDNA were independent of age and fasting plasma glucose levels in the first trimester.
- These results were in plasma samples collected 3 to 4 months before overt hyperglycemia diagnosis suggestive of GDM was made.
- Blood samples for mtDNA analysis can be collected from prenatal women irrespective of fasting status.

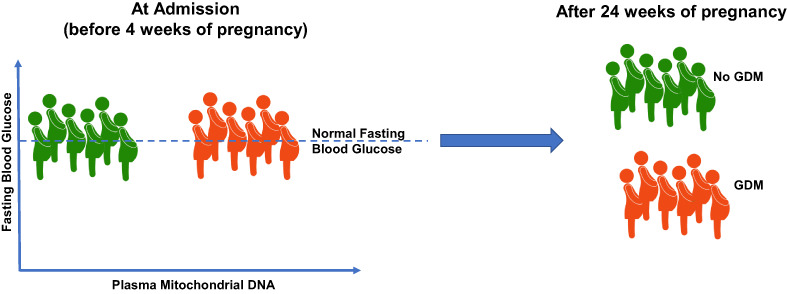

## 摘要

背景:我们评估了孕早期血浆线粒体DNA (mtDNA) 片段的丰度是否可以预测妊娠糖尿病(GDM)的风险

方法:这是一项前瞻性研究，招募了150名处于孕早期的女性。在孕早期和孕中期进行口服葡萄糖耐量试验(OGTT)诊断GDM

结果:在我们的队列中，23名女性在孕早期被诊断为GDM并被排除在本研究之外。其余的127人中，29人在孕中期被诊断为GDM，98名在整个孕期未患GDM的女性作为对照组。我们从每个参与者孕早期抽取的血液中扩增了三个不同的mtDNA基因序列:COX、ND4和D‐loop。以ΔCt方法在mtDNA和18S rRNA之间进行估计，发现每个mtDNA序列的丰度与孕中期晚期或孕晚期早期的GDM发生相关。对照组和孕中期GDM发生组之间的ΔCt COX差异有统计学意义(p=.006)。这些水平与孕早期的年龄或空腹血浆葡萄糖无关。ΔCt COX可以预测GDM，敏感度为90%，特异度为40%。虽然ΔCt ND4在GDM阳性组中较高，但差异水平未达到统计学意义。ΔCt D‐loop在GDM阳性病例和未在孕期发生GDM的对照组之间相似

结论:这些结果来自在确诊GDM之前3至4个月收集的血浆样本。血浆mtDNA片段的丰度代表了一种有前景的符合成本效益的、方便的早期生物标志物，可用于预测GDM的发展。重要的是，无论受试者是否空腹，都可以使用这一指标。需要在大型、多样化人群中进一步评估这些生物标志物的预测能力，以便有效地应用于临床

## 1 INTRODUCTION

Gestational diabetes mellitus (GDM) is a frequent and significant complication of pregnancy, characterized by glucose intolerance, insulin resistance, and hyperglycemia in the pregnant woman.^1^ It is associated with higher morbidity and mortality, both for the mother and the baby. GDM increases the risk for hypertensive disorders in pregnancy and preterm delivery and a high risk for type 2 diabetes later in life for the mother. Children born to women with GDM have the risk of excess growth during the fetal period and have an increased risk for obesity and cardiometabolic disorders later in life.^2^ Therefore, from a clinical and public health perspective, prevention and possibly early diagnosis and treatment of GDM may be vital in decreasing subsequent adverse effects involving maternal and offspring health.

There is no consensus on the screening process or appropriate cut‐off values for diagnosing GDM. According to a widely accepted recommendation of the American Diabetes Association (ADA) and the International Association of the Diabetes and Pregnancy Study Groups, GDM is diagnosed via an oral glucose tolerance test (OGTT) performed with 75 g of glucose after overnight fast at 24–28 weeks. GDM is diagnosed when one of the following cutoff values: fasting plasma glucose values of 95 mg/dL (5.1 mmol/L), 180 mg/dL (10.0 mmol/L), or 155 mg/dL (8.5 mmol/L) at 1 or 2 h, respectively.^3^ This advice is based on the assumption that most physiological insulin resistance related to pregnancy has developed by 24–28 weeks of gestation.^4^

Numerous evidence supports the adverse influence of hyperglycemia on fetal growth in early pregnancy.^5^ It can cause epigenetic changes in the fetus, making them susceptible to several long‐term pathologies.^6^ Evidence also suggests that fetal complications of in utero exposure to maternal hyperglycemia start before the traditional diagnosis of GDM from 24 weeks gestation.^7^ Therefore, some clinics have started advising OGTT even in the first trimester, that is, within the first 3 months of pregnancy. This has resulted in identifying GDM in at least 17% of pregnant women. But for those who test negative for GDM by OGTT in their first trimester, nearly 23% become positive for GDM by 24–28 weeks of gestation.^8^ So, it is reasonable to identify this subcategory of women diagnosed with GDM only toward 24–28 weeks of gestation in their early pregnancy.

Insulin resistance and hyperglycemia are elusive in the early stages of GDM, and, therefore, diagnosis is delayed until hyperglycemia is evident. Increased oxidative stress is considered essential in pancreatic β‐cell dysfunction, insulin resistance, and hyperglycemia leading to GDM.^9^ So, biomolecules that undergo a dynamic change in the presence of oxidative stress may help detect the high oxidative stress preceding clinical GDM presentation (seen as a rise in blood sugar). Although limited evidence is available about the molecular triggers for these events, the results suggest that damage to mitochondrial DNA (mtDNA) may be a necessary consequence of increased oxidative stress.^10^ mtDNA is particularly prone to oxidative damage due to its proximity to the electron transport chain, lack of a histone protective shield covering the mtDNA, and limited DNA repair mechanisms.^11^ This ultimately promotes apoptosis, which causes elevation of mtDNA levels in the blood. Hence, increased circulating cell‐free mtDNA levels in the blood may indicate oxidative cell damage, which precedes clinical GDM presentation. A previous study has shown increased plasma mtDNA in individuals with type 2 diabetes. The authors measured fragments of mtDNA known as mtDNA damage‐associated molecular patterns (DAMPs), which are released into the circulation upon cell death.^12^ DAMPs are thought to have a role in the inflammation and pathogenesis of many diseases.^13^ By extrapolating these observations to pregnant women, it is reasonable to assume that women with normal OGTT in early pregnancy who develop GDM later on should have higher levels of circulating cell‐free mtDNA before the manifestation of impaired OGTT. Estimating plasma cell‐free mtDNA is simple, and its abundance can be an inexpensive oxidative stress index.

Identifying high‐risk women for proper follow‐up and reducing obstetrical complications and adverse fetal outcomes associated with GDM are essential. Therefore, in this study, we aimed to investigate whether the first‐trimester plasma cell‐free mtDNA levels are a prognostic marker for GDM. Our results provide the first primary evidence that increased plasma cell‐free mtDNA fragments in the first trimester can predict GDM risk.

## 2 RESEARCH DESIGN AND METHODS

### 2.1 Study population

This prospective study enrolled 150 women in the first trimester of their pregnancy. All patients received regular antenatal care in the Department of Obstetrics and Gynaecology, AIIMS‐Raipur, India. The following women were excluded from the study: women with type 1 diabetes mellitus or type 2 diabetes mellitus preconception, overweight pregnant women with body mass index > 25 before pregnancy, and women with a history of GDM in previous pregnancies. GDM was diagnosed by administering OGTT at the time of their first antenatal visit (corresponding to the first trimester). Women who had normal OGTT at the first visit were screened with a repeat OGTT at the end of the second trimester or the beginning of the third trimester. ADA guidelines were used for GDM diagnosis. Accordingly, the women were considered to be GDM positive if, upon 75 g of OGTT, any one value of plasma glucose values were met: ≥95 mg/dL in the fasting sample or ≥180 mg/dL in 1‐hour plasma sample or ≥155 mg/dL in 2 h plasma sample.^3^ The Institutional Ethical Committee of AIIMS‐Raipur approved the study.

### 2.2 Biochemical measurements

Glucose was measured on a Beckman AU5800 Clinical Chemistry Analyzer in the biochemistry laboratory of AIIMS‐Raipur.

### 2.3 Sample collection for mtDNA estimation

We collected an extra vial of blood in a purple vacutainer containing EDTA at the time of collecting blood for fasting OGTT in the first trimester. This prevented multiple pricks in the woman for sample collection. Plasma was separated by centrifugation and stored at −80°C in aliquots until analysis.

### 2.4 Quantification of plasma mtDNA

Plasma circulating cell‐free DNA was isolated using QIAamp DNA Blood Mini Kit (Qiagen) from plasma aliquots (200 μL) following manufacturer protocol. The amount and purity of cell‐free DNA in samples were quantified using NanoDrop® spectrophotometry. Primers and real‐time polymerase chain reaction conditions, as described by Yuzefovych et al,^14^ were used to amplify plasma mtDNA fragments and nuclear DNA, 18S rRNA. mtDNA sequences within ND4, COX, and D‐loop genes were amplified. Plasma cell‐free mtDNA abundance was measured by the ΔCt method between Ct values of mtDNA and 18S rRNA. We correlated GMD occurrence in the late second or early third trimester with the abundance of each mtDNA sequence.

### 2.5 Statistical analysis

All statistical analyses were performed using SPSS 21.0 (SPSS Inc., Chicago, USA). Wilcoxon‐Mann–Whitney *U* Test was used for nonparametric comparisons. Analysis of variance (ANOVA) was used for parametric comparisons. DeLong's test was used to determine area under the receiver operating characteristics curve (AUROC) to evaluate the screening performance of each plasma mtDNA fragments. Because the study included only pregnant women, gender was not included in statistical comparisons. *p* values ≤ .05 were considered statistically significant.

### 2.6 Data and resource availability

All data pertaining to the results are included either in main text or as Supplementary material.

## 3 RESULTS

### 3.1 Patient characteristics

Because this was a pilot study, we calculated the sample size based on a previously published formula, *n* = *Z* ^2^ *P*(1 − *P*)/*d* ^2^, where *n* is the sample size.^15^ *Z*, the standard normal variate was taken as 1.96 at 95% confidence interval (CI). *P*, the expected prevalence obtained from a previous study, was taken as 10.77%,^16^ and allowable error, *d*, was taken as 5%. Accordingly, we enrolled 150 randomly consenting pregnant women coming to the hospital for antenatal care. Among the cohort of 150 subjects, 23 individuals were found to have GDM in the first trimester. They were excluded from the study. Of the remaining 127, 29 women were diagnosed with GDM in the second trimester. Thus, in our study group, nearly 23% of women who tested negative for GDM in the first trimester by OGTT became GDM positive by the end of the second trimester. The women who did not develop GDM during their pregnancy (*n* = 98) were controls in our study. For comparisons, the samples were divided into two groups (1): GDM in the second trimester and (2) no GDM during pregnancy or controls.

### 3.2 Correlation of first‐trimester fasting blood glucose with GDM occurrence

We compared first‐trimester fasting blood glucose between those who developed GDM only by the late second or early third trimester and those who did not develop GDM (controls). The plasma glucose values were not normally distributed. So, we did a nonparametric test (Wilcoxon‐Mann–Whitney *U* Test) to make group comparisons. The mean (SD) of fasting first‐trimester blood glucose (mg/dL) in the control group was 88.24 (8.60). The mean (SD) of fasting first‐trimester blood glucose (mg/dL) in the GDM in the second‐trimester group was 91.03 (9.72). The mean blood sugar in the GDM in the second‐trimester group was slightly higher than controls, but their difference was insignificant (*p* = .171) (Table 1, Figure S1). Also, the values of mean blood sugar in the GDM in the second‐trimester group were not high enough to be categorized under GDM.

**TABLE 1:** Comparison of the gestational diabetes mellitus (GDM) in the second‐trimester group with controls, in terms of the firstyrimester fasting blood glucose (mg/dL).

First‐trimester blood glucose (mg/dL) (fasting)	GDM at second trimester		*p* value
	Yes (*n* = 29)	No (*n* = 98)	
Mean (SD)	91.03 (9.72)	88.24 (8.60)	.171
Median (interquartile range)	89 (84–97)	89 (82–93)	
Range	75–109	66–120	

### 3.3 Correlation of age with GDM occurrence

We compared GDM occurrence in the two groups in terms of age. Because age was not normally distributed, the comparison was made using nonparametric tests (Wilcoxon‐Mann–Whitney *U* Test). The mean (SD) of age (years) in the GDM in the second‐trimester group and controls was 28 (3.97) and 26.72 (3.55), respectively. Although slightly higher in GDM positive group, there was no significant difference between the two groups in terms of age (*p* = .121) (Table 2; Figure S2).

**TABLE 2:** Comparison of the gestational diabetes mellitus (GDM) at second trimester group with controls, in terms of age (years).

Age (years)	GDM at second trimester		*p* value
	Yes (*n* = 29)	No (*n* = 98)	
Mean (SD)	28.00 (3.97)	26.72 (3.55)	.121
Median (interquartle range)	28 (25–31)	26.5 (24–29)	
Range	20–35	20–35	

Next, we divided the ages of the patients in the study into three subgroups to see the prevalence of GDM occurrence with age. Accordingly, we categorized the period into three subgroups: 20–25, 26–30, and 31–35 years. Association between “group” and “age” was done using the chi‐square test. There was no significant difference between the various groups in terms of distribution of age (*p* = .294) (Table S1).

### 3.4 Comparison of GDM occurrence with ∆Ct COX

**FIGURE 1:**
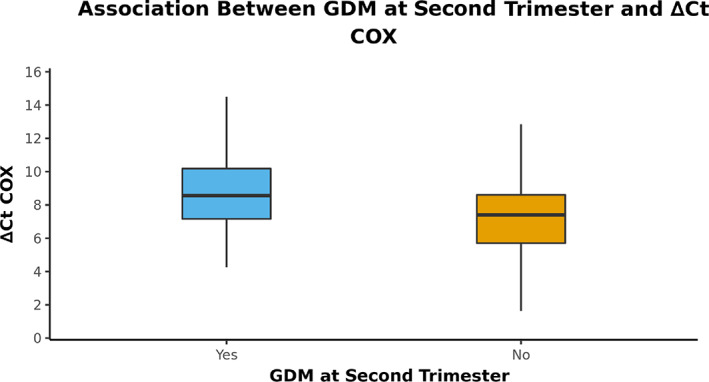
The box‐and‐whisker plot depicts the distribution of ∆Ct COX in the two groups. GDM, gestational diabetes mellitus.

The variable ∆Ct COX was not normally distributed in the two groups. Thus, nonparametric tests (Wilcoxon‐Mann–Whitney *U* Test) were used for group comparisons. A pairwise comparison between the controls and GDM in the second‐trimester group showed a significant association (*p* = .006) (Table 3). Figure 1 shows the box‐and‐whisker plot depicting the distribution of ∆Ct COX in the two groups.

**TABLE 3:** Comparison of the gestational diabetes mellitus (GDM) at second trimester group with controls, in terms of ∆Ct of various mitochondrial DNA sequences.

Gene	Sample	Mean (SD)	Median (interquartile range)	Range	*p* value
**∆Ct COX**	**GDM (*n* = 29)**	10.70 (5.84)	8.57 (7.16–10.18)	4.25–23.44	**.006**
	**Control (*n* = 98)**	8.07 (4.58)	7.41 (5.71–8.6)	1.63–23.44	
**∆Ct ND4**	**GDM (*n* = 29)**	10.15 (3.93)	9.12 (8.23–10.3)	5.59–22.16	.06
	**Control (*n* = 98)**	8.77 (3.50)	8.62 (6.84–9.62)	0.32–23.89	
**∆Ct D‐loop**	**GDM (*n* = 29)**	7.62 (2.05)	7.88 (6.43–8.99)	2.59–12.69	.56
	**Control (*n* = 98)**	7.35 (2.41)	7.46 (6.01–8.85)	1.41–15	

*Note*: Bold indicates statistically significant value (*p* ≤ 0.05).

### 3.5 Comparison of GDM occurrence with ∆Ct ND4

**FIGURE 2:**
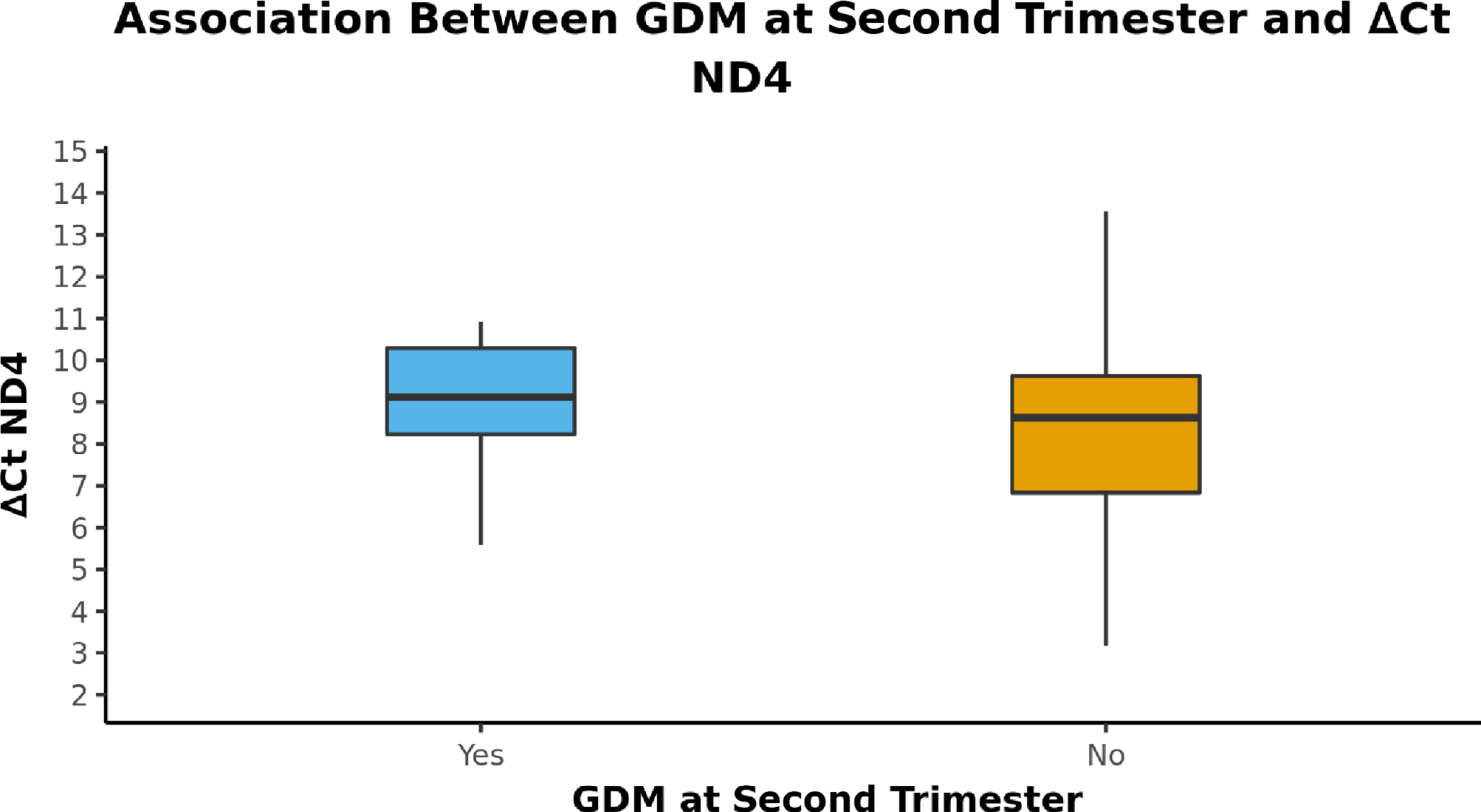
The box‐and‐whisker plot depicts the distribution of ∆Ct ND4 in the two groups. GDM, gestational diabetes mellitus.

The variable ∆Ct ND4 was not normally distributed in the two groups. Thus, nonparametric tests (Wilcoxon‐Mann–Whitney *U* Test) were used for group comparisons. Although the median among the groups was higher in the GDM in the second‐trimester group, there was no significant difference between the GDM‐positive group and controls in terms of ∆Ct ND4 (*p* = .06) (Table 3). Figure 2 shows the box‐and‐whisker plot depicting the distribution of ∆Ct ND4 in the two groups.

### 3.6 Comparison of GDM occurrence with ∆Ct D‐loop

**FIGURE 3:**
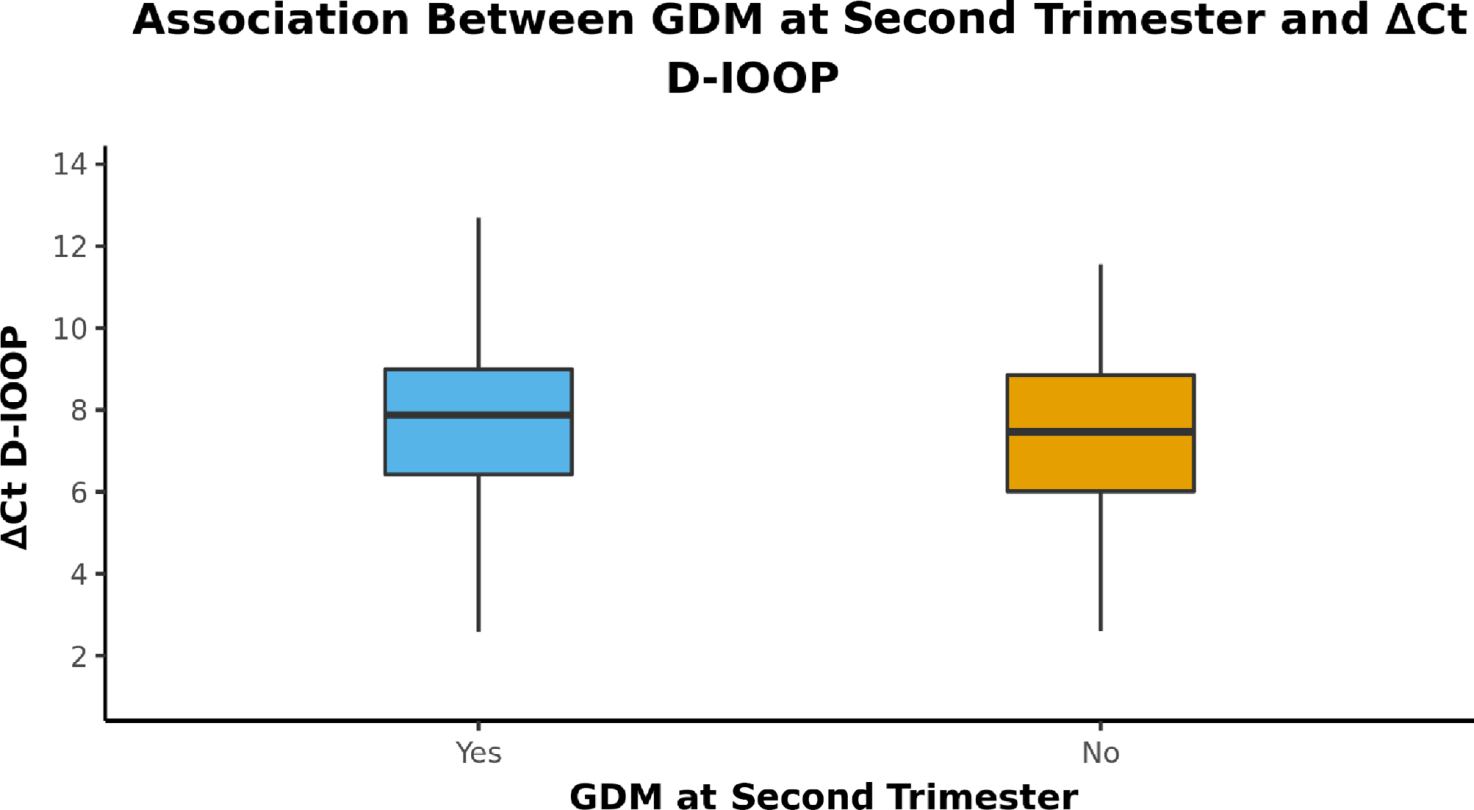
The box‐and‐whisker plot depicts the distribution of ∆Ct D‐loop in the two groups. GDM, gestational diabetes mellitus.

The variable ∆Ct D‐loop was normally distributed in the two groups. Thus, group comparisons were done using parametric tests (ANOVA). We found no significant difference between the groups controls and GDM in the second rrimester (*p* = 0.560) (Table 3). The box‐and‐whisker plot depicting the distribution of ∆Ct D‐loop in the two groups is shown in Figure 3.

### 3.7 Prediction of GDM based on all three gene sequence

**FIGURE 4:**
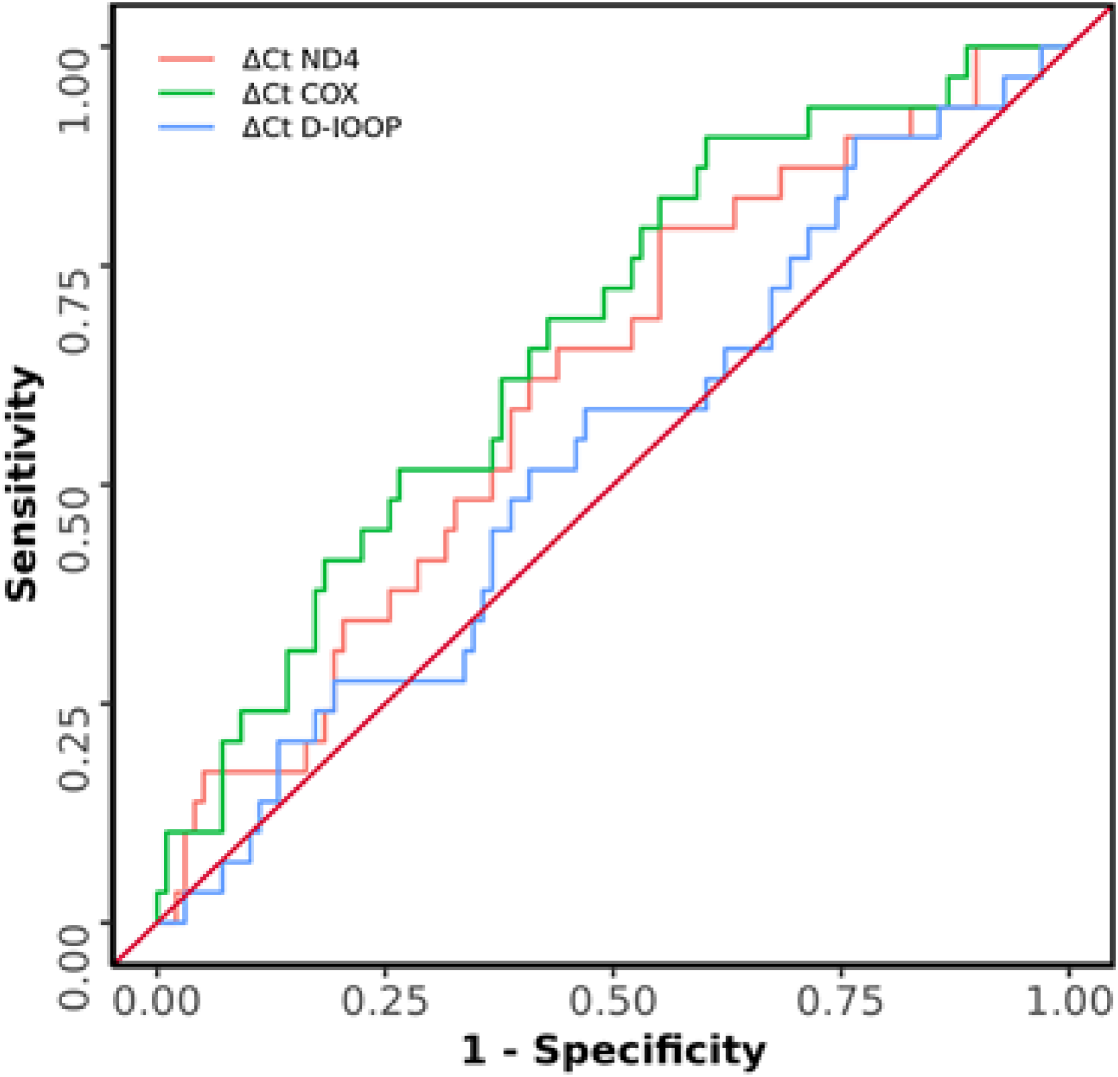
Receiver operating characteristic curve for prediction of gestational diabetes mellitus in the second trimester based on ΔCt of ND4, COX, and D‐loop.

We compared parameters for predicting GDM based on the AUROC (Figure 4). The AUROC for ∆Ct COX predicting GDM in the second trimester vs control was 0.669 (95% CI: 0.561–0.777), thus demonstrating poor diagnostic performance. However, it was statistically significant (*p* = .006). At a cutoff of ∆Ct COX ≥6.376, it predicted GDM in the second trimester with a sensitivity of 90% and a specificity of 40%. The AUROC for ∆Ct ND4 predicting GDM in the second trimester vs controls was 0.615 (95% CI: 0.502–0.729), thus demonstrating poor diagnostic performance. It was not statistically significant (*p* = .060). At a cutoff of ∆Ct ND4 ≥ 8.184, it predicted GDM in the second trimester with a sensitivity of 79% and a specificity of 45%. The AUROC for ∆Ct D‐loop predicting GDM in the second trimester vs controls was 0.536 (95% CI: 0.418–0.654), thus demonstrating poor diagnostic performance. It was not statistically significant (*p* = .556). At a cutoff of ∆Ct D‐loop ≥ 5.853, it predicted GDM in the second trimester with a sensitivity of 90% and a specificity of 23%. However, the cutoff and the diagnostic parameters of ∆Ct ND4 and ∆Ct D‐loop may not be reliable as the test is not statistically significant (Table 4).

**TABLE 4:** Diagnostic performance of first‐trimester ∆Ct of various mtDNA sequences in predicting GDM occurrence in the second trimester.

Parameter	Value (95% CI)		
	∆Ct COX	∆Ct ND4	∆Ct D‐loop
Cutoff (*p* value)	≥6.376 (.006)	≥8.184 (.060)	≥5.853 (.556)
AUROC	0.669 (0.561–0.777)	0.615 (0.502–0.729)	0.536 (0.418–0.654)
Sensitivity	89.7%	79.3%	89.7%
Specificity	39.8%	44.9%	23.5%
Positive predictive value	30.6%	29.9%	25.7%
Negative predictive value	92.9%	88.0%	88.5%
Diagnostic accuracy	51.2%	52.8%	38.6%
Positive likelihood ratio	1.49	1.44	1.17
Negative likelihood ratio	0.26	0.46	0.44
Diagnostic odds ratio	5.73	3.12	2.66

Abbreviations: AUROC, area under the receiver operating characteristics curve; CI, confidence interval; mtDNA, mitochondrial DNA.

## 4 DISCUSSION

Our study provides the first evidence that first‐trimester plasma levels of mtDNA fragments could be used as a biomarker for increased GDM risk during pregnancy. We show that first‐trimester plasma levels of mtDNA fragments are elevated in a group of pregnant women 3–4 months before the diagnosis of GDM in the late second trimester or early third trimester and that there is a significant positive correlation between elevated levels of first‐trimester plasma mtDNA fragments and GDM occurrence.

Though the exact etiology of GDM is unknown, several studies have suggested that increased oxidative stress might be a unifying mechanism promoting insulin resistance and hyperglycemia leading to GDM occurrence during pregnancy. Few studies have examined the utility of various serum oxidative markers as a predictor of GDM. In a study of 62 Thai women (30 in GDM and 32 in the control group), the maternal serum oxidative stress marker, 8Isop, and an inflammatory marker, tumor necrosis factor alpha (TNF‐α), were significantly higher in the GDM group. However, the samples were collected at 24–28 weeks of gestation, that is, end of the second or early third trimester.^17^ In another independent study, the plasma levels of advanced oxidative protein products and protein carbonyl, both markers of protein oxidation and 8‐iso‐prostaglandin F2α, a marker of lipid peroxidation, were elevated at 16–20 weeks in women who had GDM compared to controls who did not develop GDM.^18^ In yet another study, serum from 60 Indian pregnant women (30 with GDM) was analyzed for proinflammatory cytokines TNF‐α, interleukin (IL)‐6, IL‐8, and antioxidants like glutathione peroxidase, superoxide dismutase, uric acid, and Bilirubin. The proinflammatory cytokine levels, except IL‐6, were significantly increased, whereas antioxidant markers were reduced considerably in the GDM group. Again, in this study the serum collected at 24–28 weeks of gestation was used for analysis.^19^ Additionally, these studies have no evidence that the elevation was in the first trimester. Because fetal damage increases with exposure to a hyperglycemic maternal environment, an ideal serum oxidative marker should be high enough to be quantified in early pregnancy.

Insulin resistance and hyperglycemia are subtle in the early stages of GDM, and diagnosis is delayed until overt hyperglycemia is evident. Although inadequate evidence is available about the molecular triggers, the results suggest that damage to mtDNA may be an important consequence of increased oxidative stress preceding GDM.^10^ Our results strengthen these hypotheses, as we show elevated levels of mtDNA in plasma collected in the first trimester from women at increased risk for GDM compared to pregnant women who never developed GDM. Further, this difference in the abundance of plasma cell‐free mtDNA was independent of age or fasting plasma glucose levels in the first trimester. These observations also suggest that pathways leading to excessive oxidative stress are hyperactive in women who are GDM negative in the first trimester but become GDM positive by the late second trimester. Identifying these pathways is essential for understanding the etiology of GDM and reducing its prevalence.

When we compared the predicting power of the three fragments of mtDNA, the ΔCt of the COX1 sequence, which flanks the “common deletion” area,^20^ had the highest predictive value for GDM in the second trimester. It also had the highest sensitivity, followed by ND4. The sequence within D‐loop did not seem to have the same predictive power. Although additional studies are required, these observations support the idea that the abundance of plasma cell‐free mtDNA in GDM may represent increased degradation and release of mtDNA sequences under oxidative stress. Though increased plasma mtDNA represents proinflammatory molecules in various experimental models,^21^, ^22^ their biological consequences in GDM are unknown. Further studies are also required to determine whether pharmacologic strategies to lower the circulatory levels of mtDNA may prevent GDM.

A major strength of our preliminary studies is that we found an elevation in plasma mtDNA in samples collected 3–4 months before the diagnosis of GDM. The test could be administered irrespective of the fasting status of the pregnant woman, thereby preventing the need for additional prenatal visits. Although mtDNA fragments within the COX1 sequence had the strongest correlation with GDM risk, followed by the ND4 sequence, the sequence within D‐loop did not show any significant correlation. It is unclear if the differences in the predictive ability of the three sequences within mtDNA were related to their selective release in circulation or due to limited sample size. Studies with larger groups should resolve these uncertainties.

## 5 CONCLUSIONS

In this pilot study, we found that women who developed GDM had higher levels of mtDNA in their blood in the first trimester. This result is especially significant as mtDNA was raised in women 3–4 months before the development of GDM by the late second trimester. Current guidelines require screening pregnant women at the end of the second trimester or the beginning of the third trimester. These recommendations are based on the premise that GTT alone cannot detect GDM until the development of overt hyperglycemia by the end of the second trimester. Interestingly, the levels of plasma mtDNA in the first trimester were independent of age and fasting plasma glucose levels. Our results justify evaluating first trimester circulating cell‐free mtDNA as a potential biomarker for predicting GDM occurrence. It is important to note that determining plasma mtDNA abundance is less costly, minimally invasive, and more convenient than OGTT and can be easily incorporated as a routine antenatal care protocol, irrespective of the fasting status of the pregnant woman. Further assessment of the predictive capacity of these biomarkers within large, diverse populations is needed for their effective clinical utility.

## AUTHOR CONTRIBUTIONS

Dushyant Kumar Sahu contributed to sample collection, gathering clinical data, analysis, and initial draft of the paper. Jessy Abraham contributed to conception, design, and conduct of the study and edited and approved the final version of the manuscript.

## FUNDING INFORMATION

This work was made possible by generous support to Dushyant Kumar Sahu from AIIMS‐Raipur to purchase reagents and other consumables required as part of research work for partial fulfillment of the degree of MD (Biochemistry).

## DISCLOSURE

The authors declare no potential conflicts relevant to this article.

## Supporting information


**Table S1.** Association between group and age.
**Figure S1.** The box‐and‐whisker plot depicting first‐trimester fasting blood glucose (mg/dL) distribution in those who developed gestational diabetes mellitus in the second trimester and controls.
**Figure S2.** The box‐and‐whisker plot depicting the distribution of age (years) in the groups.
