# Supplementary material for: Plasma mitochondrial DNA is elevated in maternal serum at first trimester and may serve as a biomarker for prediction of gestational diabetes mellitus
Source: J Diabetes. 2023 Sep 1;15(12):1095–102. doi: 10.1111/1753-0407.13462 (PMC10755614; doi:10.1111/1753-0407.13462)

Supplementary Table 1: Association Between Group and Age

| **GDM at 2nd Trimester** | **Age** | | | | **P Value** |
| --- | --- | --- | --- | --- | --- |
|  | **20-25 Years** | **26-30 Years** | **31-35 Years** | **Total** |  |
| Yes | 8 (18.2%) | 13 (21.7%) | 8 (34.8%) | 29 (22.8%) | 0.294 |
| No | 36 (81.8%) | 47 (78.3%) | 15 (65.2%) | 98 (77.2%) |  |
| Total | 44 (100.0%) | 60 (100.0%) | 23 (100.0%) | 127 (100.0%) |  |

Supplementary Figure 1: The Box-and-Whisker plot depicting First Trimester Fasting Blood Glucose (mg/dL) distribution in those who developed GDM in the Second Trimester and controls.


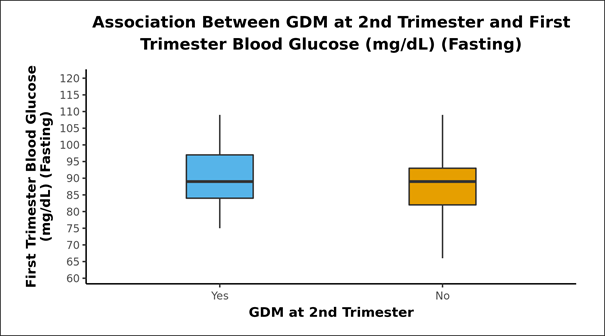


Supplementary Figure 2: The Box-and-Whisker plot depicting the distribution of Age (Years) in the groups.


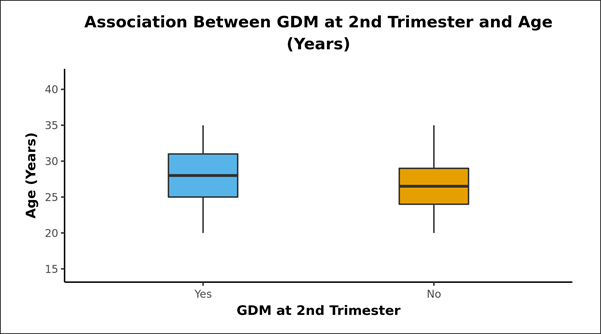

Supplement: Supplementary file 1 — Table S1. Association between group and age. Figure S1. The box‐and‐whisker plot depicting first‐trimester fasting blood glucose (mg/dL) distribution in those who developed gestational diabetes mellitus in the second trimester and controls. Figure S2. The box‐and‐whisker plot depicting the distribution of age (years) in the groups. [file JDB-15-1095-s001.docx]
